# Differential T Cell Levels of Tumor Necrosis Factor Receptor-II in Children With Autism

**DOI:** 10.3389/fpsyt.2018.00543

**Published:** 2018-11-20

**Authors:** Paul Ashwood

**Affiliations:** Department of Medical Microbiology and Immunology, and The Medical Investigation of Neurodevelopmental Disorders Institute, University of California, Davis, CA, United States

**Keywords:** autism, lymphocyte, T cells, TNF-tumor necrosis factor, behavior, cytokine receptors

## Abstract

Autism spectrum disorders (ASD) are characterized by impairments in verbal and non-verbal communication, in social interactions, and often accompanied by stereotypical interests and behaviors. A role for immune dysfunction has long been implicated in ASD pathophysiology, behavioral severity, and co-morbidities. The pro-inflammatory cytokine tumor necrosis factor alpha (TNFα) has been associated with ASD in some studies but little is known about its receptors. There are two receptors for TNFα, with TNFRI relaying many of the signals from TNFα, especially those that are rapid, whilst TNFRII relays later more long-term effects of TNFα. Proteolytic cleavage can lead to the soluble versions of these receptors which can neutralize the effects of TNFα. Here, we determined levels of TNFα and its receptors in 36 children with a confirmed diagnosis of ASD and 27 confirmed typically developing (TD) controls, 2–5 years-of-age. Children with ASD had higher levels of TNFRII on T cells compared to controls following cell stimulation. Levels of sTNFRII were decreased in cell supernatants following stimulation in ASD. Overall these data corroborate the role of inflammatory events in ASD and align with previous studies that have shown differential changes in cellular adaptive immunity in children with ASD. Future longitudinal analyzes of cellular immune function and downstream signaling from immune receptors will help further delineate the role of inflammation in ASD.

## Introduction

Autism spectrum disorders (ASD) are neurodevelopmental disorders affecting 1 in 59 children whom are characterized by significant deficits in communication, social interactions and frequently accompanied by stereotyped or restricted behaviors and interests. The etiology of ASD is complex and largely unknown; however, potential genetic candidates linked with ASD include many genes that regulate immune responses, for instance human leukocyte antigen (HLA)-DR, phosphatase and tensin homolog (PTEN), macrophage migration inhibitory factor (MIF), complement C4B, MET tyrosine receptors, interleukin (IL)-4 receptor, and reelin [Reviewed in (1, 2)]. Significant immune dysfunction is also seen in children with ASD, including prominent neuroinflammation in brain specimens, and alterations in adaptive and innate immune responses in the periphery (3–15).

Tumor necrosis factor-α (TNFα) is a pleiotropic and highly regulated pro-inflammatory cytokine secreted by a number of different cells that plays an important role in coordinating early inflammatory processes, cell proliferation and apoptosis (16). Biologically the activities of TNFα are mediated via two different ubiquitously expressed TNFα receptors, TNF receptor type I (TNFRI; also known as CD120a), and type II (TNFRII; CD120b) (17, 18). TNFRI seems to be the main mediator of TNFα rapid signaling and is found on most tissues, whereas TNFRII mediates later more long-term effects of TNFα and are more commonly expressed on immune cells (17, 19). Proteolytic cleavage of these receptors from the cell surface results in soluble forms (sTNFRI and sTNFRII) that can neutralize TNFα and thus modulate its biological activity (20). The measurement of levels of sTNFRs and TNFα together may be more useful and reliable markers of the inflammatory response and TNFα bioactivity than just TNFα alone.

Although many studies have documented increased expression of TNFα in different neuropsychiatric diseases, for example schizophrenia, depressive disorder, and Alzheimer's disease, only a few have evaluated TNFα in serum/plasma or following stimulation of immune cells in ASD, and the results are often conflicting (3, 4, 21–36). Information on the levels of sTNFRs are even more scant in ASD. Finally, no previous study has assessed both levels of TNFRs on the cell surface of immune cells and production of sTNFRs in ASD.

The present study sought to evaluate levels of TNFRs on T cells and sTNFRs in supernatants following immune challenge in ASD patients compared to TD control subjects.

## Methods

### Subjects

This study examined 63 participants enrolled through the Childhood Autism Risk from Genetics and Environment (CHARGE) study at U.C. Davis (37). Full details regarding behavioral measures/assessments and recruitment in the CHARGE study protocols have previously been described (37). Children were consecutively assessed. Participants were free of medication and without chronic clinically defined illness or fever at time of blood draw. The participants were 27 typically developing (TD) controls median age 3.9 years [(interquartile range 2.2–6.1), 4 females] and 36 children with ASD [median age 3.6 years (interquartile range 2.5–4.8), 5 females]. Diagnoses of ASD was based on Diagnostic and Statistical Manual of Mental Disorders, Fourth Edition (DSM-IV) criteria, and defined as autistic disorder. Further evaluation was confirmed using the Autism Diagnostic Interview-Revised (ADI-R) and the Autism Diagnostic Observation Schedule (ADOS) assessments. Children from the TD groups were screened for autism traits using the Social Communication Questionnaire (SCQ). This study was approved by the UC Davis institutional review board and complied with all requirements regarding human subjects. Parents gave both written and informed consent.

### Cell isolation, stimulation, and biochemical measures

Peripheral blood mononuclear cells (PBMC) were separated from the whole blood by centrifugation over Histopaque-1077 Hybri-Max lymphocyte separation medium (Sigma; St. Louis, MO) before washing twice in Hanks Balanced Salt Solution (HBSS; VWR; Brisbane, CA). PBMC were either cultured in media alone, or stimulated with PHA (10 μg/mL; Sigma), for 24 h at 37°C in 5% CO_2_. Following culture, plates were centrifuged before supernatants were harvested and stored at −80°C until cytokine analysis and cells processed for flow cytometry.

The quantification of TNFα and its soluble receptors (sTNFRI and sTNFRII) were assessed by ELISA using standard procedures recommended by the manufacturer (Quantikine, R&D Systems, Minneapolis, Minn., USA). All samples were on unstimulated and stimulated cell culture supernatants, in duplicates. The detection limits for the kits were < 0.5 pg/ml. Concentrations obtained below the sensitivity limit of detection (LOD) of the method were calculated as LOD/2 for statistical comparisons. Culture supernatants had not undergone any previous freeze/thaws cycle.

Cells were harvested after culture and were washed three times in FACS buffer (PBS, 1% fetal bovine serum albumin (VWR, USA) and 0.1 % sodium azide (Sigma), before being resuspended and stained in 100 μl FACS buffer containing either the following monoclonal antibodies fluorescein isothiocyanate (FITC)-conjugated mouse anti-human TNFRI (CD120a); phycoerythrin (PE)-conjugated mouse anti-human TNFRII (CD120b); (PE)-Cy5-conjugated mouse anti-human CD3; and allophycocyanin (APC)-conjugated mouse anti-human CD4, CD8 (all antibodies were from BD Biosciences, CA, USA). Appropriate IgG isotype controls (BD bioscience) were used to correct for compensation issues. Cells were incubated at 4°C for 30 min before being spun down and washed with staining buffer. Cells were then analyzed on a LSR II flow cytometer and the data acquired analyzed with FlowJo software (BD Immunocytometry Systems). Lymphocytes were gated using forward scatter and side scatter parameters and CD3^+^ cells for analysis of cell surface TNFRI and TNFRII expression, with further analysis of CD4 and CD8 expression where each parameter was measured separately on CD3^+^ populations, CD3^+^CD4^+^ populations, and CD3^+^CD8^+^ populations.

### Statistical analysis

In primary analyses, induced TNFα and soluble receptors and cell surface markers levels (outcome) were compared by group (predictor) and statistical significance was determined using a parametric Student's *t*-test, following confirmation of normal distribution, with a *p*-value of < 0.05 considered significant. Multiple comparisons were adjusted for by using the Benjamini-Hochberg False Discovery Rate. Using answers to questions regarding loss of language (Q11) and loss of social skills (Q25) of the ADI-R, the autism population was further divided into two groups based on the clinical onset of autistic symptoms; namely, children who regressed in acquired language or social skills after initial typical development, and secondly, children who did not regress. We also compared ADOS scores with immune outcomes using Pearson correlations. There were too few subjects with clinical co-morbid features such as gastrointestinal symptoms or sleep disorders to determine differences between cytokine production or cell surface receptor expression. All analyses were carried out using SAS version 9.1 (SAS Inc.; Cary, NC) and graphed with Prism 5 Software (GraphPad Software; San Diego, CA).

## Results

Cytokine and soluble receptors were measured in harvested supernatants from unstimulated and PHA-stimulated, PBMC cultures. No difference between children with ASD and TD controls were observed in TNFα or its receptors in unstimulated media alone conditions (Table 1). Activation with PHA led to an increase in all analytes measured across both groups. Observed levels of sTNFRII after PHA-stimulation were significantly less in ASD group compared to the TD group (*p* = 0.014; Table 1). When comparisons were made among children with ASD who had regressed compared to those that had not, unstimulated sTNFRII levels were lower in children with ASD who had not regressed, compared to those that had and to TD controls (*p* < 0.04). However, following PHA-stimulation, both groups were decreased for sTNFRII compared with TD controls and were not significantly different from each other. No other differences were observed in ASD children based on regression. Levels of sTNFRII were significantly negatively correlated with impairments in ADOS social interactions (*r* = −0.383, *p* < 0.025), suggesting lower levels were associated with worse behaviors.

**Table 1:** Comparison of the TNFα and soluble receptors (pg/ml) following cell culture in media alone or stimulation with PHA in children with autism (*n* = 36) and typically developing controls (*n* = 27). Data are presented as mean ± standard error of means (SEM).

		**TNα**	**sTNFRI**	**sTNFRII**
Media	ASD	34.38 ± 2.81	57.80 ± 2.13	147.7 ± 13.07
	TD	33.09 ± 3.844	58.23 ± 2.70	182.4 ± 22.80
PHA	ASD	54.50 ± 1.89	63.83 ± 2.48	1, 060 ± 77.72
	TD	54.71 ± 1.96	66.17 ± 3.49	1, 766 ± 213.61^*^

* *p = 0.014 stimulated levels of sTNFRII were decreased in ASD children compared with PHA stimulated levels in typically developing controls*.

**Figure 1:**
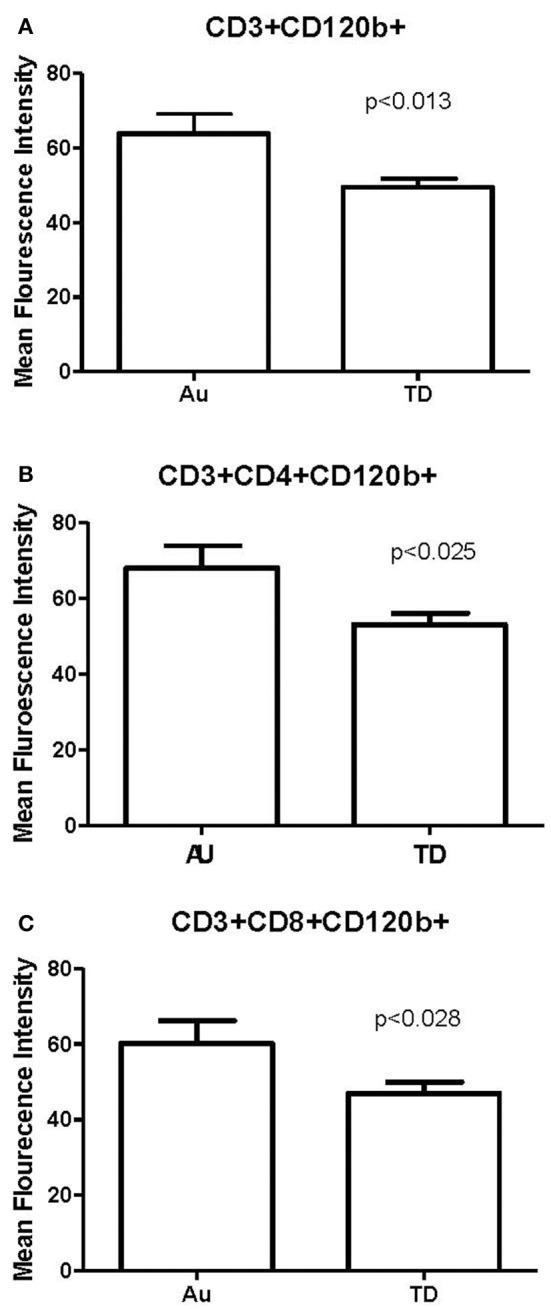
Comparison of CD120b (TNFRII) on T cells and T cell subsets following PHA stimulation in children with autism (Au: *n* = 36) and frequency and geographically matched typically developing (TD; *n* = 27). Data are expressed as a Mean Fluorescence intensity (MFI) of CD120b on **(A)** CD3^+^ T cells, and **(B)** CD3^+^CD4^+^ and **(C)** CD3^+^CD8^+^ T cell subsets. Data is represented as Mean and standard error of mean, exact *p*-values included.

No significant differences in the frequencies of CD3^+^, CD4^+^, and CD8^+^ T cells between TD controls and children with ASD, with or without PHA stimulation were observed. The frequency of TNFRI expressing or TNFRII expressing T cell subsets was not different between groups in unstimulated or stimulated conditions (data not shown). Following immune stimulation both the frequency of cells expressing TNFRI (CD120a) and TNFRII (CD120b) on the cell surface were similar in both groups. However, after PHA stimulation, the amount of cell surface TNFRII (CD120b) receptors, as measured by mean fluorescence intensity (MFI), was significantly increased in children with ASD compared to controls on CD3^+^ T cells as a whole, and both the T helper CD3^+^CD4^+^CD120b^+^ subsets and cytolytic CD3^+^CD8^+^CD120b^+^ T cells (*p* < 0.03; Figure 1). No differences were seen between those children with ASD who had regression and those children with ASD that did not have regression. In the ASD group frequency of CD3^+^CD120b^+^ were associated with worse social behavior on ADOS (*r* = 0.238, *p* < 0.035).

## Discussion

To the best of our knowledge this is the first study to assess levels of TNFRs on immune cells and release of sTNFR into cell culture supernatants following immune challenge in ASD. Our results demonstrated increased levels of TNFRII on T cells and T cell subsets following stimulation, and decreased sTNFRII in supernatants in children with ASD, but no differences in TNFα and cell surface or sTNFRI levels were found.

Moreover, we found associations between TNFRII levels on cells or in the supernatants and more impairments in behavior. It is currently unclear how TNFα can affect neurodevelopmental outcomes and behaviors during childhood in ASD, and the data should be treated with caution. However, of note, numerous studies have shown that impairments in core ASD behaviors and associated co-morbid and aberrant behaviors, are strongly correlated with altered immune profiles (38). Further validation of the link between observed behavioral severity and cytokine and cytokine receptors is warranted.

Multiple studies have demonstrated that TNFα levels are increased in serum of individuals with ASD when compared to controls (21, 23, 25, 26, 34–36); however, studies on plasma have provided conflicting results (4, 28, 35). These discordant results may reflect the matrix used as well as methodological issues, including different assessment instruments, and clinical and demographic characteristics of ASD populations studied. In addition, it is possible that TNFα is produced at affected tissues and degraded shortly after its production. As TNFRs can be induced by TNFα, the cellular expression may relate to TNFα activity. Our data suggest an increase in TNFRII on T cells, with subsequent decreases in supernatants presumably due to decreased shedding. Previous studies by Jyonouchi and colleagues corroborate our findings showing decreased sTNFRII in cell culture supernatants after T cell mitogen stimulation with PHA (29–32); however, cell surface expression of TNFR was not previously determined. Soluble TNFRII is generated by proteolytic cleavage by the metalloproteinase TNFα converting enzyme (TACE, also known as ADAM17) which despite this enzyme being increased in blood of ASD children (39) does not result in increased sTNFRII in our study, perhaps suggesting proteolytic cleavage is not the underlying mechanism and that receptors are retained on cells. Another possibility is that cell signaling after TNFα binding is weak and that proteolysis does not occur due to altered signaling pathways (40). Soluble TNFRII may lead to the inactivation of circulating TNFα by the generation of high affinity complexes. These complexes reduce the binding of TNFα to any cell membrane target receptors thus downregulating the activity and response to TNFα (41). As soluble TNFRs neutralize TNFα, measuring both receptor and cytokine may be more reflective of the net effect of cytokines, in that even though we did not see an increase in TNFα after stimulation, the increased presence of receptors on the cell surface and decreased levels of neutralizing sTNFRII in supernatants may indicate that more TNFα-receptor ligand binding and signaling occurred. Future studies should determine TNFα-TNR responses in immune cells from children with ASD, including other immune cell types not just T cells. Further studies should also address whether cytokines such as TNFα are involved in pro-apoptotic signaling or pro-life signaling and the net balance of those signals induced by inflammatory responses in ASD.

Differential expression of markers of T cell activation have been shown in children with ASD following immune stimulation including increased CD26, CD38, CD69, HLA-DR, but decreases in CD25 (42–46). Another member of the TNF receptor family, CD137 is a co-stimulatory molecule expressed by activated T cells and enhances T cell proliferation, effector functions, and survival was also increased on T cells in ASD children (43). TNFRII is predominantly found on immune cells and is primarily associated with lymphocyte proliferation (17, 19). The TNFRs differ in their intracellular domains which induce distinct intracellular signals. We recently showed that T cell signaling was altered in children with ASD (40) and may suggest that certain downstream signals such as the mTOR pathway may lead to preferential immune activation. Taken together these results may suggest potential differential activation of T cell subsets in ASD when compared to controls.

In summary, the results presented here are in agreement with prior studies and suggest that inflammatory processes, as evinced by alterations of pro-inflammatory cytokine signaling, could contribute to ASD pathophysiology. It is also possible that other aspects of ASD might provide additional feedback that influences or compounds the altered immune state in ASD. Further, future studies including longitudinal measurements of the same participants might further reveal the putative role for levels of TNFRs as bio signatures of ASD development and progression. More studies will be required to characterize the changes in TNFα and the TNRII and their temporal relationship with ASD development, as well as assessment of cytokine and cognate receptors in the brain.

## Author contributions

The author confirms being the sole contributor of this work and has approved it for publication.

### Conflict of interest statement

The author declares that the research was conducted in the absence of any commercial or financial relationships that could be construed as a potential conflict of interest.
